# Insufficiency in functional genomics studies, data, and applications: A case study of bio-prospecting research in ruminant microbiome

**DOI:** 10.3389/fgene.2022.946449

**Published:** 2022-08-31

**Authors:** Kgodiso J. Rabapane, Grace N. Ijoma, Tonderayi S. Matambo

**Affiliations:** College of Science, Engineering, and Technology, Institute for the Development of Energy for African Sustainability (IDEAS), University of South Africa, Roodepoort, South Africa

**Keywords:** bioprospecting, microbial ecology, functional genomics, ruminant, biocatalysis, ’omics technology

## Abstract

Over the last two decades, biotechnology has advanced at a rapid pace, propelled by the incorporation of bio-products into various aspects of pharmaceuticals, industry, and the environment. These developments have sparked interest in the bioprospecting of microorganisms and their products in a variety of niche environments. Furthermore, the use of omics technologies has greatly aided our analyses of environmental samples by elucidating the microbial ecological framework, biochemical pathways, and bio-products. However, the more often overemphasis on taxonomic identification in most research publications, as well as the data associated with such studies, is detrimental to immediate industrial and commercial applications. This review identifies several factors that contribute to the complexity of sequence data analysis as potential barriers to the pragmatic application of functional genomics, utilizing recent research on ruminants to demonstrate these limitations in the hopes of broadening our horizons and drawing attention to this gap in bioprospecting studies for other niche environments as well. The review also aims to emphasize the importance of routinely incorporating functional genomics into environmental metagenomics analyses in order to improve solutions that drive rapid industrial biocatalysis developments from derived outputs with the aim of achieving potential benefits in energy-use reduction and environmental considerations for current and future applications.

## Introduction

Microorganisms are ubiquitous (Coughlan et al., 2015), and they are also uniquely found in certain extreme environmental conditions referred to as niches. Certain microorganisms tend to become richer in biodiversity than others due to the interdependent relationship between the habitat constitution and the accompanying microbial behavioral adaptations necessary to derive nourishment from available substrates and ultimately sustain life under these mostly harsh conditions. This epistemology has yielded a pragmatic research approach since finding novel organisms and products derived from them is most likely higher in these niches (extreme environments) than in mundane habitats (Sysoev et al., 2021). The gastrointestinal tract (GIT) of ruminants represents such a niche rich in biodiversity due to the mechanism by which various bio-products are derived.

Ruminants are mammals exhibiting an evolutionary but unique digestive system comprised of a multi-chambered stomach with diverse microorganisms. These autochthonous symbiotic microbial consortia influence the breakdown of the mainly lignocellulosic feed (indigestible by monogastric animals) obtained from their herbivorous diet. The synergistic microorganisms convert the feed into a wide array of metabolites such as volatile fatty acids (VFAs), amino acids, and others required by the ruminants for metabolic activity, physiological growth, and overall health (Puniya et al., 2015). The microorganisms involved in the fermentation of feed in the ruminants’ GIT include mainly bacteria, archaea (comprising mainly of methanogens), and Eucarya (protozoa and fungi). In general, the majority of bacteria that have been identified are from the *Bacteroidetes*, *Firmicutes*, and *Proteobacteria* phyla (Malmuthuge et al., 2015; Smoliński et al., 2021). Additionally, microbial constitution and carbohydrate-active enzyme (CAZyme) profiles were observed in a buffalo’s GIT metagenome study. Moreover, a higher abundance of oligosaccharide degrading and debranching enzymes, cellulose, and hemicellulose degrading enzymes has also been observed (Patel et al., 2014).

Other unique ecological niches and extreme environments, such as hot water springs, hydrothermal vents, salt pans, and oil-contaminated soils, have proven to be potential sources for microbial populations that have adapted genetic traits to secrete various biomolecules biomolecules (enzymes, whole cells, refined pathways, bioactive compounds e. t.c) (Cowan et al., 2015; Lauro and Williams 2015). Pharmaceuticals, biotechnology, bioremediation, aquaculture, bioenergy, nanotechnology, and agriculture all benefit from these traits and products (Coughlan et al., 2015; Lauro and Williams, 2015). As a result, an organized biological exploration of these niche environments, including ruminants’ GIT, to achieve an enhanced bioprocess and ease of process optimization (scale-up) for commercialization in order to save time, energy, and cost while meeting the population’s needs is a credible justification for bioprospecting (Sawarkar et al., 2019; Pooja and Narayanapur, 2022). Thus, the overarching motivation for bioprospecting ruminants’ GIT is to discover organisms from which unique and high-titer volumes of bio-products can be extracted. Furthermore, concerted efforts to ensure the consistent application of desired products in industries must include a clear understanding of the genetic sequences and functions of these microbial communities (genotype-phenotype relationships) (Rexroad et al., 2019).

Several studies on the GIT of ruminants have so far primarily focused on the cornerstone of natural classification with resolutions, sometimes to the species level, that rely heavily on the traits of phylogenetic markers found in ribosomal RNA and genes coding for rDNA (Nyonyo et al., 2014; Djurhuus et al., 2017; Potprommanee et al., 2017; Iqbalsyah et al., 2019). This quick approach may be tunnel-visioned, as it is primarily focused on phylogenetic classification of microbial diversity and the ability to delineate species based on evolutionary gaps using 16S ribosomal genes for bacteria and archaea (Kim and Chun 2014; Denman and Mcsweeney, 2015), 18S for high-resolution taxonomic eukaryotes, and internal transcribed spacer regions (ITS) for fungal environmental samples (Kim and Chun 2014; Denman and Mcsweeney, 2015; Badotti et al., 2017). Although this method allows for the identification and tracking of microbial species, as well as the identification of novel groups of microorganisms whose function and significance within an environment are unknown, such information, while they are useful, they serve little purpose in advancing bioprocess applications. It neither provides a comprehensive overview of individual microorganisms’ interspecific interactions nor biochemical contributions to ruminant GIT microbial populations. To address these limitations, advances in molecular biology, particularly in the field of “omics technology” (genomics, metagenomics, transcriptomics, proteomics, and metabolomics), combined with Next Generation Sequencing (NGS) platforms, have made significant contributions to bioprospecting high-throughput (HTP) microbial consortia genome-genome interactions at the phenotypic level, with the goal of mining for isozymes and novel enzymes (Ribeiro et al., 2016). Furthermore, omics technology, specifically functional genomics using NGS, reveals knowledge that defines phylogeny and diversity, the roles these microbial communities play in their natural environments, and how they can be applied for industrial and commercial purposes in the future.

Functional genomics research in niche environments has significantly increased our understanding of the impact it potentially has in accelerating the bioprospecting of novel bio-products. However, the pace witnessed in the medical and pharmaceutical industries is by no means comparable to that in other industries and environmental bioremediation programs, both of which are dismally lagging. Several factors, including the complexity of sequence data analysis, may be considered a deterrent (Chistoserdova, 2010; Mande et al., 2012; Teeling and Glöckner, 2012; Sharpton, 2014; Ghurye et al., 2016). Perhaps, the perspective that these aspects of bioprospecting for industry and the environment may be regarded as strictly commercial pursuits compared to medicine and pharmaceuticals, where there are more obvious links to virtuous research linked to human health, may also contribute to the reduced focus. However, the new pattern clarifies that the focus on reshaping industrial and environmental processes through the application of biologically derived products will definitely contribute to a reduction in environmental pollution and indirectly benefit the overall well-being of humans.

The scarcity of functional genomics studies associated with the GIT potentially provides a microcosm towards understanding limitations experienced with other promising niche environments that would have benefited from in-depth investigations and the concomitant benefits such knowledge would provide to relevant industries. Therefore, this review hopes that by highlighting these challenges specific to ruminant functional genomic studies, attention will be drawn to the new pragmatic shift that should begin in our quest to integrate ‘omics technologies into research and development for industrial biocatalysis. The quantitative genetic studies executed provided a scope to explore the predicted roles of the microorganisms identified. In the past, ruminants’ microbiomes have been used as whole cells in Microbial Fuel Cells (MFCs) to generate electricity from cellulose (Rismani-Yazdi et al., 2007; Wang et al., 2012). Thus, studying the functions of these genomes can result in the sustainable utilization of structural plant material (lignin). However, the phylogenetic marker and culture-based methods have been identified as limiting factors in exploring the microbiome of ruminant origin due to the fastidious and anoxic environment predominant in the GIT of ruminants and the difficulty of replicating these exact conditions in bench studies. Consequently, only an estimated 11% of the ruminant microbial community has been cultured, leaving the uncultured, 89%, as a mystery that is yet to be revealed. So, this review will also discuss the important role of culturomics and the limitations of cultivation methods as a way to learn more about the fastidious ruminant microbiome (Zehavi et al., 2018).

In the past, cost restrictions were one of the significant limitations to conducting functional genomics studies since they involve whole-genome sequencing. However, with advancements, there has been a noticeable but marginal decrease, which has eased the availability of genome sequences, but not enough to be impactful, especially with the ruminants’ GIT microorganisms. Notably, researchers in the ruminant community have initiated the Hungate1000 project that aims to sequence 1,000 genomes from ruminant microbiomes, based on the acknowledged lack of genome-genome data in this niche environment. Moreover, the field of bioinformatics is faced with the reality that the majority of genes encode proteins without apparent specific roles (Blaby and Blaby-haas, 2017), making correlations to functions rather difficult. Thus, the arduous task of separating and identifying specific genes that have been assigned roles or functions amidst the milieu of genomes will continue to confound scientists for many years.

Nonetheless, a broader perspective on the ruminant microbiome and its underlying functions through a genome catalog such as the Hungate1000 promises the implementation of strategies for feedstock formulation, digestion efficiency, methane abatement, and contribution towards environmentally friendly approaches to several adaptable industrial biocatalytic processes. Hence, in this review, we will cover the advent of functional genomics as it bridges the gap between quantitative and qualitative genetics, function, and its application to transcriptomics, proteomics, and metabolomics. Furthermore, we explore how functional ‘omics unravels layers of data as it relates to gene-specific roles while generating a broader perspective of microbial physiology under various conditions and parameters, the current status of the ruminants’ microbial genomes in connection with the Hungate1000 project, and how ruminant microbial genome-system studies can provide protein function inferences within ruminants, either for feedstock formulation and/or improvement, methane abatement, or for industrial purposes as a whole.

## The gastrointestinal tract of ruminants as a functional niche environment

### The digestive process of ruminants as a source of microorganisms

Ruminants have a multi-chambered stomach comprising four compartments (rumen, reticulum, omasum, and abomasum) (Hungate, 1947). The multi-chambered stomach is a distinctive trait that differentiates them uniquely from monogastric animals. The foregut digestive system in ruminants is an adaptive feature to accommodate the ruminal symbiotic microorganisms that are required for feed fermentation since they lack the hydrolytic enzymes necessary for the breakdown of the plant structural carbohydrates such as lignin, cellulose, and hemicellulose, which form a significant part of their diet as herbivores (Jewell et al., 2015). The actual microbial fermentation of the ingested feed occurs in the largest of all the compartments, the rumen. Small particles are transferred from the rumen and the reticulum to the omasum. However, large and potentially digestible feed particles are forced back up (regurgitation) through the esophagus to the mouth for further particle size reduction through chewing (rumination) (Hungate, 1947). The insignificant separation between the rumen and the next compartment, the reticulum, plays a crucial role in rumination and makes it easy for the feed to pass through. Together, the rumen and the reticulum have a size-capacity for feed and fluid that ranges from 50 to 120 L (Moran, 2005), depending on the type of ruminant. This large capacity easily accommodates the large volumes of saliva secreted during grazing. It is primarily involved in the rumination process and enables microorganisms to form biofilms by attaching to feed particles, making it feasible to access and initiate anaerobic fermentation. The secreted saliva also plays a crucial role as a buffering agent by releasing salts, mainly bicarbonate, and regulates the physiological pH range between 5.5 and 6.9 (Choudhury et al., 2015) during the microbial fermentation of feed, which results in the production of VFAs (butyric, propionic acid, and acetic acid) in excess amounts at varied ratios depending on the type of feed.

Once the mechanical and alkaline pre-treatment has reduced the feed particle size, it flows to the next compartment, the omasum, where the VFAs produced during fermentation are absorbed. Finally, different enzymes and acids are released to aid in digestion in the last compartment, the abomasum, known as a true stomach, akin to that found in monogastric mammals (Hungate, 1947; Russell, 2002). Microorganisms carried with the digesta from the rumen are digested in the abomasum and form part of the diet. This digestion of microorganisms enables ruminants to extract energy from cellulose and microbial protein efficiently. The digesta then flows to the small intestines and later to the large intestines of the ruminant, where further digestion occurs (Hungate, 1947; Russell, 2002; Moran, 2005). Table 1 represents the taxonomic suborder Ruminantia (ruminants) encompassing six different families, Tragulidae, Antilocapridae, Giraffidae, Cervidae, Moschidae, and Bovidae, with the Bovidae being the most explored niche environment for various microorganisms and enzymes (Chen et al., 2019).

**TABLE 1:** Taxonomic family, subfamily and species common name of ruminants

Family	Subfamily	Species common name
Trangulidae	-	Lesser mouse deer
Antilocapridae	-	Pronghorn
Giraffidae	-	Okapi and Giraffe
Cervidae	*Capreolinae*	Roe deer, Reindeer and White-tailed deer
	*Cervinae*	White-lipped deer, Milu
	*Muntiacinae*	Chinese muntjac, Indian muntjac, Black muntjac
Moschidae	-	Forest musk deer
Bovidae	*Bovinae*	African buffalo, Cattle, Yak, Lesser kudu, Common eland, Greater kudu, Bushbuck, Sitatunga, Mountain nyala, Bongo
	*Aepycerotinase*	Impala, Suni
	*Antilopinae*	Klipspringer, Royal antelope, Kirk’s dik-dik, Steenbok, Przewalski’s gazelle, Oribi, Thomson’s gazelle, Grant’s gazelle, Gerenuk, Springbok
	*Cephalophinae*	Maxwell’s duiker, Harvey’s duiker, Common duiker
	*Reducinae*	Bohor reedbuck, defassa waterbuck
	*Hippotraginae*	Gemsbok
	*Alcelaphinae*	Blue wildebeest, Topi, Hartebeest
	*Pantholopinae*	Tibetan antelope
	*Capridae*	Argali, Sheep, Barbary sheep, Blue sheep, Ibex, Goat

Adapted from Chen et al. (2019).

Some of the most common microorganisms in the GIT of ruminants are bacteria (10^11^ cells/mL), methanogens (10^6^ cells), fungi (10^3^–10^6^ zoospores/mL), protozoa (10^4^–10^6^ cells/mL), and bacteriophages (10^9^ particles/mL) (Hungate, 1947; Han et al., 2015; Ribeiro et al., 2016). These microorganisms play various unique roles in bringing about the complete anaerobic digestion of the overall feed. Bacteria and protozoa are primarily involved in the digestion of starch and fibrolytic feed, while gut fungi are responsible for the efficient degradation of fiber in the GIT. At the same time, methanogens actively utilize hydrogen to complete an anaerobic fermentation process that results in methanogenesis. The regulated pH range (6.2–7.0) at rest, and the maintained temperature range of 38–42°C, adhesion, moisture content, ionic strength, and redox potential, among other factors (Malmuthuge et al., 2015; Hill et al., 2016; Huang et al., 2018), allow for anaerobes and facultative anaerobes to proliferate in the ruminant’s GIT resulting in a diversity of microorganisms of which only about 11% (Zehavi et al., 2018) have so far been cultivated.

### Role of major microorganisms in the ruminant GIT and their potential contribution towards economic value

The dominant phyla in the ruminants’ GIT are *Bacteriodetes*, *Firmicutes*, *Proteobacteria,* and *Actinobacteria* (Smoliński et al., 2021)*.* The central aspect that defines the composition of microbial communities in the GIT is diet. Ruminants that feed profusely on fibrolytic components (lignin, cellulose, and hemicellulose) harness an increased lignocellulosic microbial population, and the optimal function of pH also becomes highly regulated at 6.0–6.8 due to the typical production of volatile fatty acids (acetic, propionic, and butyric acid) (Ososanya et al., 2013). In comparison, ruminants that feed on a high-grain diet experience altered pH and microbial composition because of the increased production of organic acids and VFAs, which cause a lowering of the pH (less than 5.5), and cumulatively, a condition referred to as sub-acute ruminal acidosis (SARA). Furthermore, once the pH in the rumen goes lower (5.0) or above 7.8, there is a likely diminished buffering capacity, causing a reduction in biodiversity and affecting the growth of protozoa and those that grow well at pH above 6.0–6.2 (fibre-digesting microorganisms) (Chiba, 2009; Faniyi et al., 2019). In a study done by Ogata et al. (2019), a high-grain diet was introduced to Japanese black cattle to increase intramuscular fat accumulation, resulting in the production of high concentration levels of lactic acid, which is more acidic than VFAs in the rumino-reticulum. The low acid conditions resulted in the abundance of bacterial genera of unclassified *Ruminococcaceae* and unclassified *Lachnospuraceae* and the genus *Intestinimonas,* which can be associated with lactic acid metabolism (Ogata et al., 2019). In addition to a change in bacterial composition and low pH in the rumino-reticulum, a lower bacterial diversity was also observed when a high-grain diet was compared with a high-fiber diet (Kim et al., 2018). Although the presence of high lactic acid and total VFAs in the rumino-reticulum may result in severe conditions due to acutely low pH, the microorganisms that utilize these acids proliferate and assist in the biomass turnover while regulating the normal pH range and sustaining the proliferation of the acid-intolerant microorganisms that act on fiber (Choudhury et al., 2015), thus maintaining the homeostatic balance of this environment. Such an example describes how the microbial population in the GIT plays a significant role as bio-networks to keep the conditions in the GIT favorable for their proliferation and, in general, the ruminant’s physiological health. Some of the main microbial species that have adapted symbiotic mechanisms towards the digestion of various feedstuffs in the ruminants’ GIT are recorded in Table 2.

**TABLE 2:** GIT microorganisms and their various roles in feedstuff degradation.

Major groups	Role	Genera and species
Bacteria	Cellulolytic	*Fibrobacter succinogenes* (Hungate, 1947; Stewart and Flint, 1989)*, Butyrivibrio fibrisolvens* (Hungate, 1950)*, Bacillus licheniformis* (Fujimoto et al., 2011)*, Ruminococcus flavefaciens* (Sijpesteijn, 1951)*, R. albus* (Hungate, 1957)*, Clostridium cellobioparum* (Hungate, 1944)*, C. chartadabidum* (Kelly et al., 1987)*, C. longisporum* (Hungate, 1957)*, C. lochheadii* (Hungate, 1957)*, Eubacterium cellulosolvens* (Taguchi et al., 2008)
	Hemicellulolytic	*Eubacterium xylanophilum* (Johns, 1951)*, E. uniformis* (Johns, 1951)
	Amylolytic	*Streptococcus bovis* (Hungate et al., 1952)*, Ruminobacter amylophilus* (Hamlin and Hungate, 1956)
	Lipolytic	*Anaerovibrio lipolytica* (Hobson and Mann, 1961)
	Proteolytic	*Prevotella ruminicola* (Wallace, 1996)*, Clostridium bifermentans* (Clarke, 1961)
	Saccharolytic	*Succinivibrio dextrinosolvens* (Bryant and Small, 1956)*, S. amylolytica, Bacteroides ruminocola* (Bryant et al., 1958)*, Selenomonas ruminantium* (Bryant, 1956)*, Lactobacillus acidophilus, L. casei, L. fermentum, L. plantarum* (Perry and Briggs, 1957)*, L. brevis, L. helveticus* (Briges, 1953)*, Bifidobacterium globosum, B. longum, B. thermophilum, B. ruminale, B. ruminantium* (Scardovi et al., 1969; Trovatelli and Matteuzzi, 1976)
	Pectinolytic	*Treponema saccharophilum* (Paster and Canale-Parola, 1985)*, Lachnospira multiparus* (Bryant and Small, 1956)
	Acid utilizers	*Megasphaera elsdeni* (Elsden et al., 1956)*, Wolinella succinogenes* (Wolin et al., 1961)*, Veillonella gazogene* (Johns, 1951)*, Micrococcus lactolytica, Oxalobacter formigenes* (Allison et al., 1985)*, Desulfovibrio desulfuricans* (Howard and Hungate, 1976)*, Desulfotomaculum ruminis* (Coleman, 1960b)*, Succiniclasticum ruminis* (Van Gylswyk, 1995)
	Acetogens	*Acetitomaculum ruminis* (Greening and Leedle, 1989)*, Eubacterium limosum* (Genthner et al., 1981)
	Tanninolytic	*Streptococcus caprinus* (Brooker et al., 1994)*, Eubacterium oxidoreducens* (Krumholz and Bryant, 1986)
	Ureolytic	*Megasphaera elsdenii* (Gutierrez et al., 1959; Marounek et al., 1989)
Protozoa		*Entodinium bovis* (Wertheim, 1935)*, E. bubalum, E. fujitai, E. tsundotai, E. ogmotoi, E. parvum* (Imai, 1981)*, E. caudatum* (Coleman, 1960a)*, E. bursa, Epidinium caudatum,* (Kamra, 2005)*, Isotricha prostoma, I. intestinalis, Dasytricha ruminantium* (Gutierrez, 1955)*, Diplodinium dendatum, D. nanum, D. africanum, Ostracodinium iwawoi, Eudiplodinium kenyensis* (Imai, 1988)*, Oligoisotricha bubali* (Dehority et al., 1983)*, Polyplastron multivesiculatum* (Ellis et al., 1989; Paul et al., 1990)*, Eremoplastron asiaticus* (Banerjee, 1955)*, E. bubalus* (Dehority, 1979)
Fungi		*Piromyces communis* (Orpin, 1977)*, P. mae* (Gaillard-Martinie et al., 1992)*, P. minutus* (Ho et al., 1993b), *P. rhizinflatus, P. spiralis* (Ho et al., 1993c)*, P. polycephalus* (Chen et al., 2002)*, Ruminomyces elegans Anaeromyces mucronatus* (Breton et al., 1990)*, A. elegans* (Ho et al., 1990)*, Caecomyces sympodialis* (Chen et al., 2007)*, Cyllamyces icaris* (Sridhar et al., 2014)*, Neocallimastix frontalis* (Braune, 1913)*, N. patriciarum* (Orpin and Munn, 1986)*, N. hurleyensis* (Webb and Theodorou, 1991)*, N. variabilis* (Ho et al., 1993a)*, Orpinomyces joynii* (Li et al., 1991)*, O. intercalaris* (Ho et al., 1994)*, O. bovis* (Barr et al., 1989)
Methanogens		*Methanobacterium formicicum* (Oppermann et al., 1957)*, M. ruminantium* (Smith and Hungate, 1958)*, M. bryantii* (Bryant et al., 1967)*, Methanobrevibacter ruminantium* (Bryant, 1965)*, Methanomicrobium mobile* (Pazur and Forstberg, 1978)*, Methanosarcina barkeri* (Jarvis et al., 2000)*, Methanoculleus olentangyi* (Skillman et al., 2004)

Gut fungi are the most efficient fiber degraders because they act on the cuticle of lignocellulosic feed, however, they are available in smaller quantities (approximately 20% microbial mass) as compared to other organisms within the biota. These lignocelluloses are gradually degraded as structural components by bacteria and ciliate protozoa. In comparison, when attacked by anaerobic fungi, these recalcitrant foods degrade quickly and easily. Bacteria play the most active role in the degradation of complex and simple carbohydrates, and they account for the majority of biomass turnover in the GIT (approximately 50%). They have been studied for xylanolytic, cellulolytic, and hemicellulolytic activity, among other things (Seo et al., 2013; Nyonyo et al., 2014). The protozoa in the GIT also engulf structural and simple carbohydrates while regulating fermentation conditions to produce acetate, butyrate, and hydrogen (H_2_). Ammonia and amino acids from bacterial protein are also end products of protozoal activity in the rumen, as they engulf bacteria in the GIT in addition to the actual feed. Protozoa also use their slow growth to consume a large number of simple carbohydrates and store them in their bodies, resulting in fewer VFAs being produced and, as a result, contributing to the pH regulation of the rumen.

Due to the complexity of the structural components of fiber, they are digested slowly. As slow growers, protozoa take advantage of this by forming an association with fibrolytic feed to ensure survival in the GIT and avoid early elimination from the tract, thereby increasing the likelihood of their proliferation and population establishment. As a result, a high-fiber diet promotes an increase in the number of viable biomass of these dependent ciliates. Carbon dioxide (CO_2_) and H_2_ are the primary end products of this anaerobic microbial interaction with feed, in addition to VFAs. Methanogenic archaea quickly use the latter to eliminate hydrogen’s inhibitory effects on fermentation while producing methane gas (CH_4_), which is then removed by eructation because ruminants have no use for it. Ruminal methanogenesis is metabolically disadvantageous as it reduces the efficiency of energy intake from feed (Haque, 2018), and also slows down overall growth and milk production.

Although, the population of methanogens varies according to the type of ruminant in question, phylogenetic studies identified the genus *Methanobrevibacter* as the largest group in the rumen of most bovine species (Whitford et al., 2001), including sheep (Wright et al., 2004; Wright et al., 2008), and cattle (Wright et al., 2007; Zhou and Hernandez-Sanabria, 2010). Thus, methanogenic archaea are extensively utilized in biogas reactors and widely studied for methane abatement strategies in a natural setting, such as ruminants, as they are one of the major contributors to global warming (about 30%) by releasing this greenhouse gas (GHG) into the atmosphere (Altermann et al., 2018). In addition, the naturally co-existing phages or viruses balance biomass turnover in the GIT through cell lysis, resulting in immediate access to amino acids for the ruminant from microbial protein. However, the mechanism or extent to which these phages affect the bacteria or the methanogens in the rumen is still largely unknown, although ongoing research (Leahy et al., 2010) indicates that their presence is of utmost importance in exploring methane abatement strategies in ruminants due to their high specificity in infecting. This interaction is pivotal, considering that the ruminal microorganisms share a mutualistic relationship with the host animal. Some of the bacteriophages or archaeaphages that are not of rumen origin but have been reported to infect methanogens are *Methanobacterium phage Ψ M1, Methanobacterium phage ΨM2, Methanobacterium phage Ψ M10, Methanobacterium phage Ψ M100,* and *Methanothermobacter phage Ψ M100* among others (Ackermann, 2007; Puniya et al., 2015)*.*

The GIT microorganisms are continuously exposed to various feedstuffs as a consequence of foraging activities to meet daily nutritional requirements. The changes in feed composition create a suitable niche environment, resulting from adaptation and novel microbial strains that secrete unique enzymes with enhanced properties involved in various degradative pathways. Moreover, microorganisms and the derivatives from this environment have “Generally Regarded as Safe” (GRAS) status, thereby encouraging their direct use in the food, pharmaceutical, and other industries. Considering the underlying advantages, it is remarkable that research in this area remains sparse. Therefore, the need to exploit these ruminal microbial populations for various enzymes of economic value is essential, as this would contribute to a great extent towards increasing our catalog of biological catalysts useful for process optimization and energy demand mitigation in several industries.

## The premise for bioprospecting enzymes in the ruminants GIT

### Major groups of enzymes that are of interest

A market analysis report published in 2020 showed that the global enzymes market was valued at USD 9.9 billion in 2019, and its estimated compound annual growth rate projection is 7.1% for 2020–2027. This is due to rising end-use demand in industries such as food and beverage, biofuel, animal feed, and detergents (http://grandviewresearch.com/industry-analysis/enzymes-industry). Microbial enzymes are the most valued and sought-after biological products because of the many benefits resulting from their utilization. High reaction specificity, food quality improvement, role in food processing and preservation, reduction in energy requirements for certain chemical processes, and the eco-friendly solutions provided by their inclusion in industrial processing steps are among the most important of their valued characteristics, as are their versatile application and ease of microbial genetic manipulation towards the scale-up production of these enzymes. Additionally, due to their specificity, enzymes reduce waste generation, which ultimately saves time and energy and lowers operating capital in the long run. The type of reaction that these enzymes catalyze plays a significant role in their industrial demand (Allied-market-research, 2018). For example, hydrolases are the most prominently applied enzymes, holding approximately two-thirds of the enzyme market share due to their usefulness in the food and beverage industries (Dublin, 2019). Other notable examples of enzymes that are in high demand include carbohydrase (cellulases, amylases, xylanases, lactases, pectinases, pullulanases, e. t.c), proteases, lipases, polymerases, and nucleases (Dublin, 2019). Table 2 shows that carbohydrase, protease, and lipase enzymes are abundantly secreted by the microorganisms in the GIT of ruminants, making the ruminants a potential source for a wide variety of these microbial enzymes in various isoforms.

In most cases, operational temperature, pH range, and an enzyme’s lack of consistency and stability can all contribute to commercialization constraints. Thus, increased efforts in bioprospecting microorganisms from harsh environmental conditions such as the GIT of various ruminants increase the likelihood of obtaining enzymes that require minimal bioengineering or cloning, which can then be improved and enhanced for unique properties. Previous bioprospecting efforts have resulted in organisms and enzymes with unique thermotolerance, halophilicity, cold resistance, and stability under a variety of other harsh conditions. For example, Gurumurthy and Neelagund (2012) investigated a geothermal spring and isolated an industrially viable extreme thermostable novel alpha-amylase from Geobacillus sp. iso5. This strain was thermotolerant and alkali-resistant, with maximum activity at 90°C and pH 8.0, respectively. In 2013, Jaouadi et al., 2013 and colleagues investigated soil contaminated with leather tannery waste and discovered an extracellular keratinase US (KerUS) isolated from the novel *Brevibacillus brevis* strain US575, which demonstrated remarkable optimal activity at pH 8.0 and 40°C. KerUS exhibited unique keratinolytic activity and consistency in degradation when used alone for dehairing rabbit, bovine, goat, and sheep hair. A study conducted by Chary and Devi (2018) on a lipase-producing strain, *Pseudomonas aeruginosa*, isolated from soil and sewage waste, revealed that this bacteria has exceptional stability in its enzyme activity at pH 7.0 to 10.0 and 45°C. This alkaline stability has the potential to be useful in the production of detergent additives, leather, and specialty chemicals.

Aside from studies that focus on optimizing the stability of enzymes in natural and extreme conditions, molecular techniques such as site-directed mutations and directed evolution are currently used to improve the attributes of microbial enzymes, which enables them to withstand the prolonged duration of reactions in large bioreactors. These strategies have been reported to increase the production of enzymes by 100-fold (Singhania et al., 2010); as such, they can also be channeled to broaden the industrial applications of the array of enzymes highlighted in Table 3. As is demonstrated by previous studies such as that executed by Li et al. (2013), they purified, characterized, and cloned a thermotolerant isoamylase produced by *Bacillus* sp. CICIM isolated from a soil sample collected from a volcanic hot spring. The cloned isoamylase displayed its optimal activity at a remarkably high temperature of 70°C and pH 6.0, with thermostability between 30 and 70°C and an alkaline pH range from 5.5 to 9.0 (Li et al., 2013). Upon doing the carbohydrate hydrolysis test, it was concluded that this isoamylase would be very useful as a debranching enzyme. Similarly, Kaur et al. (2016) cloned a gene encoding extracellular lipase from the *Bacillus licheniformis* strain isolated from an Indian hot spring. The lipase gene was expressed in *E. coli* BL21 and displayed activity at a broad range of pH (9.0–14.0) and temperature (30–80°C). This recombinant enzyme further exhibited approximately 100% activity in the presence of isopropanol and methanol, with ∼60–90% in mixtures with acetone and toluene.

**TABLE 3:** Industrially relevant enzymes that can be derived from ruminants.

Industry	Enzyme	Role/Function	Ref
Food and Beverage	Lipase, Protease, amylase, cellulase	Texture and quality improvement; increase product shelf-life	(Kuhad et al., 2011; Andualema and Gessesse, 2012; Verma et al., 2012; Robinson, 2015; Jayasekara and Ratnayake, 2019; Razzaq et al., 2019; Singhal et al., 2020)
Detergents	Cellulase; lipase; protease	Enhance color brightness; stain removal; anti-redeposition of ink particles	(Kuhad et al., 2011; Andualema and Gessesse, 2012; Verma et al., 2012; Zhang and Zhang, 2013; Robinson, 2015; Singh et al., 2016; Jayasekara and Ratnayake, 2019; Razzaq et al., 2019)
Biofuel	Lipase; amylase; Cellulase; xylanase	Bioconversion of polysaccharides biomass	(Kuhad et al., 2011; Verma et al., 2012; Jayasekara and Ratnayake, 2019)
Textile	Cellulase	Improves absorbance capacity of fibres, fabric quality and firmness; biostoning of jeans and biopolishing of textiles fibres; soften garmets; remove excess dye; restore color brightness	(Kuhad et al., 2011; Zhang and Zhang, 2013; Jayasekara and Ratnayake, 2019)
Leather	Protease	Degradation of non-collagenous materials of the skin; non-fibrillar proteins removal	(Andualema and Gessesse, 2012; Singh et al., 2016; Razzaq et al., 2019)
	Lipase	Degreasing for fat removal; enzymatic wash and denim treatment	
Paper and pulp	cellulase; xylanase	Drainage and enzymatic deinking improvement; Co-additives in pulp bleaching and biomechanical pulping; increases fiber brightness and strength properties; increase biodegradability; reduces viscosity	(Kuhad et al., 2011; Andualema and Gessesse, 2012; Zhang and Zhang, 2013; Jayasekara and Ratnayake, 2019)
	Amylase	Improves whiteness; minimizes pollution in wastewaters and enhances pitch control; removal of triglycerides and waxes	
	Lipases		
Waste water treatment	Xylanases; cellulase; pectinase; amylase	Hydrolysis of cellulosic and starch waste	(Kuhad et al., 2011; Verma et al., 2012; Singh et al., 2016; Razzaq et al., 2019)
	Protease	Degrade poultry waste; hair epilation, unclogging of organics from pipes and drainage	
	Lipases	Treatment of residual water and effluent contaminated by oil particles; degrade organic debris and sewage from versatile activities; thin layered fat removal from aerated tanks’ surfaces	
Agriculture	Cellulase	Protect plants from biological stress; improves generation of protoplasts in fungi and plants; seed germination; improved root system and plant growth as well as quality of soil	(Kuhad et al., 2011; Verma et al., 2012)
	Lipases	Synthesize organic compounds for use as herbicides/pesticides	
Animal and feed	Xylanase; cellulase	Improve nutritional quality, dietary inclusions to maximise nutrient absorption; improves fodder quality through preservation	(Kuhad et al., 2011; Zhang and Zhang, 2013; Razzaq et al., 2019)
	Lipase	Digestibility of lipids	
	Protease	Modification of feed quality; enhance flavour, solubility and digestibility; reduce allergenic compounds	
Diagnostics	Amylase	Digestive disorders	Tiwari et al. (2015)
	Proteases	Development of effective therapeutic agents (clot-dissolving, anticancer, anti-inflammatory, antimicrobial)	
	Lipase	Clinical diagnostic tools for the quantitative determination of health disorders; digestive ailments; high cholesterol levels	
Pharmaceutical	Xylanases; Proteases	Production of prebiotics and anti-inflammatory agents	Singh et al. (2016)
Research and Biotechnology	Amylase	Additional approach for selecting successful recombinants	Singh et al. (2011)
	Protease	Assist cells to carry chemical reactions	
Personal care and Cosmetics	Lipases	Production of esters; generation of higher quality products	(Andualema and Gessesse, 2012; Verma et al., 2012)
Organic synthesis	Lipases	Design novel drugs; biosurfactants; bioactive compounds; oleochemicals	Andualema and Gessesse, (2012)

In cases where a strain is well-known to produce a sufficient amount of a certain enzyme, its system is developed in a way conducive to overproducing that particular enzyme. A notable example is the fungus *Trichoderma reesei,* known for its stable expression and production of cellulases. Studies have alluded to enhancing the hydrolytic efficiency of this fungus by employing aspects of genetic modifications to the versatile cellulase system of *T. reesei*. It should be noted that the emphasis on these three groups of enzymes mentioned in the previous sections is driven by the food and beverage industry as they are in high demand for brewing and baked products (Kaul and Asano, 2012; Gurung et al., 2013; Adrio and Demain, 2014; Vester et al., 2014), which are staple processed foods in almost all countries around the world. Table 3 highlights some of the versatile uses of carbohydrases, proteases, and lipases.

As much as the versatility of microbial enzymes, specificity, uniqueness, and the ease of bulk production are established, the extensive exploration of these microbial enzymes from the ruminants’ GIT, especially those with enhanced and novel catalytic and stability under competitive conditions, remains a mystery due to data and database insufficiency on the sequence and linked function as a result of some of the limitations that will be discussed in this review.

## Limiting factors that contribute to insufficient sequence-function data of the ruminants’ GIT microbiome

### Traditional culture-dependent methods

The conventional or classical culture-based methods have been the cornerstone of studying the ruminant’s GIT microbiome since 1947 (Hungate, 1947). It has brought about extensive insight into some of the roles of microorganisms and physiological characterization related to feed flow and nutrient availability in the GIT of ruminants. However, this method is considered limiting in its approach, as it validly contributes to the insufficiency of sequence and function data on the ruminant GIT. This culture-dependent method involves the isolation of pure cultures from environmental samples for further analysis, which routinely includes morphological, microscopic, and biochemical characterization and the comparison of results with databases for similarities that facilitate identification. Although a critical assessment has shown that microscopic analyses can sometimes be biased because the presumption of a single colony representing a cluster of identical cells is often disproved upon close inspection under the microscope, wherein the varied structures of the non-homogeneous cell shapes will be visible. These discrepancies are difficult to resolve just by mere visual observations on growth plates.

Furthermore, there is a likelihood of false reactions in testing for biochemical traits, which is a consequence of multiple chemical reactions and responses from the varied organisms interacting. Thus, the guarantee of axenic culture representation on plates is primarily based on an individual’s technical mastery of microbiology culture techniques. Moreover, some genera such as *Bacillus*, *Gemella*, and *Listeria* and certain Gram-positive anaerobes would appear peculiar upon performing Gram staining with reactions showing as Gram variable or negative when viewed under the microscope creates a characterization bias (Franco-Duarte et al., 2019). Identifying microorganisms using these conventional methods is laborious and time-consuming, with process time ranging from 2 to 5 days or more depending on the growth rate of the organism and the type of tests to be undertaken (Franco-Duarte et al., 2019). In addition, the traditional approach of classifying microorganisms also employs the use of the well-known *Bergey’s Manual*. However, a large number of microorganisms could not fit into any of the recognized taxonomic scales, ultimately rendering these phenotypic methods incompetent due to an underestimation of microbial diversity in a given sample by failing to identify closely related microorganisms at the species level or at the strain level (Klijn, 1996; Franco-Duarte et al., 2019).

Nevertheless, the culture-dependent methods and the phenotypic properties have provided tangible insights into ruminal studies since their inception. It is also essential to include that some microorganisms are unable to grow under controlled conditions due to their low prevalence and their reliance on other organisms within a consortium to create the conditions necessary for them to thrive; as such, only these interspecific interactions guarantee their proliferation and stability of such microbial populations (Köpke et al., 2005; Vartoukian et al., 2010). Moreover, their fastidious characteristic nature is a direct consequence of the environmental conditions found uniquely within the ruminant’s digestive system, such as varying pH, temperature, gradually progressive anoxic conditions, and the continuous exposure to various feed compositions due to seasonal variations or grazing based migrations. These changing conditions contribute to the constant evolution of microbial populations, and therefore, simulating such conditions within a laboratory environment tends to be a difficult challenge (Zehavi et al., 2018). Consequently, culture-based methods are considered somewhat limited for the extensive exploration of these non-culturable but viable strains of ruminant GIT origin. This trend is not peculiar to the GIT environment but has been observed in most extreme environments, and the outcomes are similar, with a resulting underestimation of the microbial diversity of a given sample. Thus far, the obscurity of the estimated 89% of the ruminant microbial community provides justification for routine exploration with its potential for industrially relevant enzymes and possible applications in various aspects of biotechnology as well as environmental mitigation processes.

### Exploring targeted hypervariable regions (16S, 18S, and ITS), cost of sequencing, and read lengths associated with the culture-independent approach

Technique advancements have included the use of molecular identification approaches, including culture-independent strategies, to alleviate culturability limitations and improve reproducibility and scalability. At first, these molecular approaches were only concerned with bridging the gap between phenotypic and genotypic trait comparisons. This technique uses nucleic acid sequences derived solely from pure cultures, allowing for the assessment of pairwise sequence similarities between two strains to provide easy strain identification for newly isolated organisms. However, the majority of the identified strains were obtained from old culture collections, and given the culture limitation of fastidious cultivations, the robustness of these molecular techniques in identifying and classifying microorganisms was limited (Kim and Chun, 2014).

**FIGURE 1:**
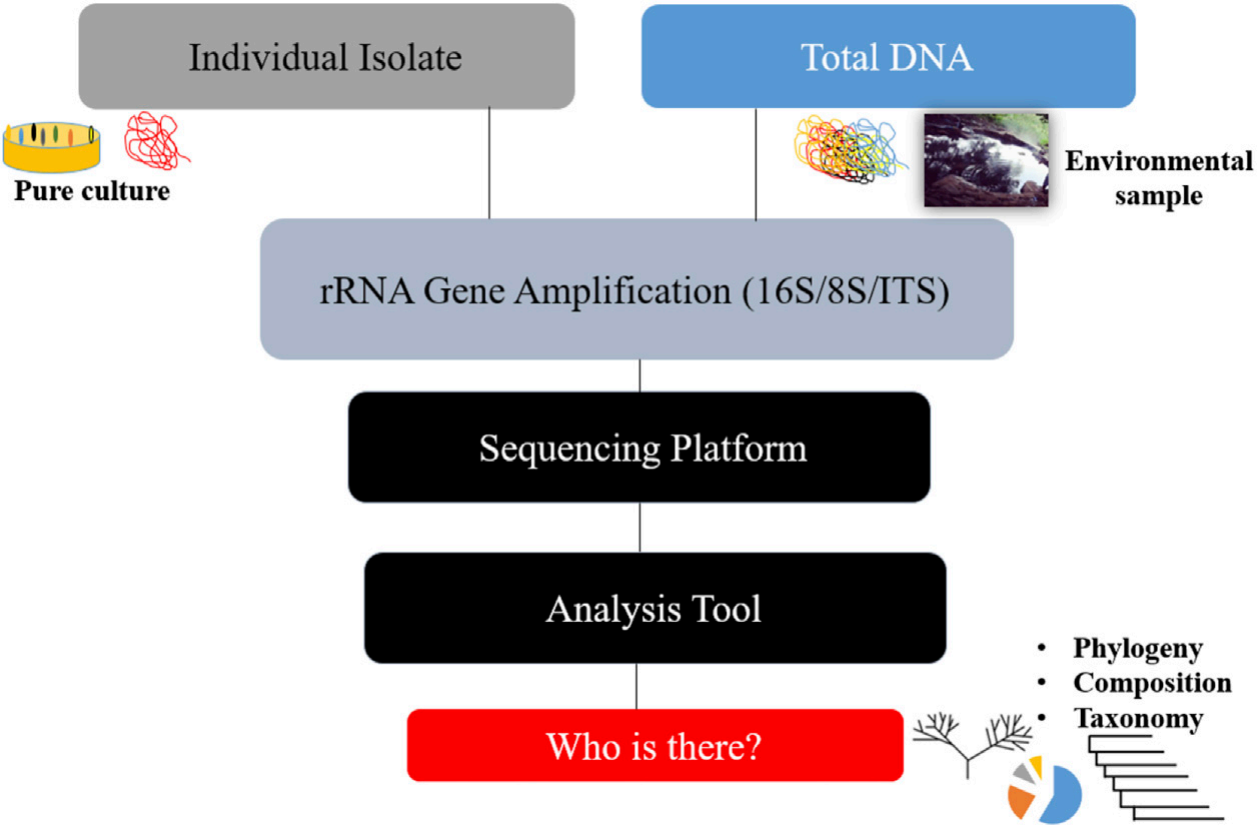
A brief illustration of the rRNA gene amplification approach and its outcome.

The apparent development that resulted from this limitation was the aggregate extraction of nucleic acids from biological and environmental samples of microbial communities in order to estimate diversity within a population and compare relative abundance across similar environmental samples. To meet the daunting challenge of dealing with the milieu of organisms within a given environment, a targeted approach that utilized hypervariable regions within different organisms’ genetic sequences to make individual identifications easier in vast populations, as is the case in all ecological environments, became necessary. In general, rRNA gene analysis methods are geared toward phylogenetic classification of microbial populations and the ability to delineate microorganisms based on evolutionary gaps via the universal 16S ribosomal gene for bacteria and archaea (Kim and Chun, 2014; Denman and Mcsweeney, 2015), 18S for high-resolution taxonomic eukaryotes, and internal transcribed spacer (ITS) regions, which are most widely used (Denman and Mcsweeney, 2015; Badotti et al., 2017). As a result, in this regard, this approach can only answer the question ‘who is there?’ in the environment depicted in Figure 1. However, such an answer only begins the inquiry process in a scientific investigation of any environment, so further investigation is required if the intention has an associated objective of biotechnology applications, which is often the ultimate goal for most bioprospecting expeditions. With the need to elucidate functional characteristics, bioinformatics software packages and repositories such as PICRUSt (Phylogenetic Investigation of Communities by Reconstruction of Unobserved States) and CowPi, which uses aspects of PICRUSt and an open-source, web-based platform known as Galaxy for data processing, have been developed. Both web-based tools aim to predict functional profiles of microbiomes based on taxonomic composition. This has resulted in the collection of sequencing data from both 16S marker genes and shotgun metagenomics.

The targeted hypervariable regions are exploited to reconstruct phylogenies based on the theory that the rate of evolution is relatively slow in these regions, and as such, they are conserved within organisms. The presence of these highly conserved RNA genes, such as the 16S, 18S, 28S, and ITS regions within prokaryotes, eukaryotes, and fungi, has made standardization for identification easy with the construction of primer sequences. These explorations of microbial diversity and identification using targeted hypervariable regions are a rapid method of identifying and compiling valuable taxonomy data and tracking various microbial species. The extent of conservation varies extensively among these hypervariable regions, with more conserved regions designating higher levels of the taxonomy (i.e., kingdom, family) and less conserved regions denoting lower levels of the taxonomy (i.e., genus and species). The revolution in microbial identification, which emanated from the ribosomal RNA gene, allows for the rapid elucidation of various genus and species from different environments, including the ruminants’ GIT, while addressing the possibility of bridging the gap between the phenotypic and genotypic traits of microorganisms. Exploring these highly conserved regions involves the generation of an amplicon library using different primer pairs that only target certain regions of the hypervariable rRNA gene. For instance, the 16S contains nine (V1–V9) different hypervariable regions with ∼30–100 base pairs (bp) (Yang et al., 2016), and various primer pairs are required for these regions depending on the aspect under investigation (Větrovský and Baldrian, 2013). One of the first studies to explore the phylogenetic diversity of the bacterial community within the rumen was done on dairy cattle using comparative sequence analysis of cloned 16S rDNA amplified from DNA extracted from ruminal fluid (Whitford et al., 1998). The cattle were fed a specific diet comprised of haylage, corn, and silage. The total DNA extracted from the rumen fluid was then subjected to two different trials. The first trial analyzed 31 cloned rRNA gene sequences in order to evaluate biases that may be introduced during the reactions; in the second trial, the amplification of parental DNA was done using either 12 or 30 cycles of PCR, and eventually, 53 (for 12 cycles), and 49 (for 30 cycles) sequences were analyzed. The existing sequences in the Ribosomal Database Project (RDP) were then compared with the sequences from the 5′end of the 16S rRNA gene from the trials. The data provided conflicting results for the cloned rRNA genes, most likely due to the presence of several novel (uncharacterized) genes and the application of an under-represented database, making credible identification nearly futile. It is also noteworthy that their study did not specify which hypervariable regions on the 16S rRNA gene were targeted; the primers used in this study are primers F27 and R1492.

The bacterial and archaeal community structure analysis in the rumen microbiome of goats by Cunha et al. (2011) using primers 27F/1492R for bacteria and primers 109F/915R for archaea demonstrated a similar trend. Their study observed that the predominant phyla from sequences of bacteria were Bacteroidetes and Firmicutes, with the overall dominant classes being Clostridia and Bacteroidia. In addition, some archaeal sequences from the phylum Euryarcheota were assigned to the *Methanobacteria* of the genera *Methanobrevibacter* and *Methanosphaera.* However, several more groups of archaea isolated from the rumen microbiome could not be assigned, and remarkably, they were grouped based on their similarities to marine bacteria represented as “uncultured marine bacteria,” specifically, groups II and III (Cunha et al., 2011). Supplementary Table S1 highlights some of the rRNA gene (rDNA) studies done on individual isolates, microbial communities, and the set of primers used in more recent times.

Although the prokaryotic 16S rRNA gene approach has historically been utilized in environmental microbiology and molecular research evolution because it is considered a reliable marker for taxonomy and phylogeny analysis of microorganisms, some studies question the sensitivity, correlation, and precision of capturing only these hypervariable regions. Yang et al. (2016) present an argument that is based on the absence of a standard approach to specific regions (i.e., V2 to V8 regions) but also emphasize the dearth of high-throughput methods to sequence full-length 16S rRNA genes, noting that it is only with such broader sequences that optimal regions can be identified for any given sample. In their case, the V4–V6 was considered the optimal sub-region for the design of universal primers. They further support their assertions regarding the apparent underestimation of the microbial population within a given sample, which eventually limits the diversity of the RDP. Simply put, correlations made based on this one aspect (16S rRNA) are considered an overreach.

Furthermore, PCR’s influence on data by bias amplifications is a credible limitation that further hampered the rRNA approach (Burke and Darling, 2016). However, opinions on the efficacy of the various hypervariable regions differ, and their suitability for phylogenetic analysis and taxonomic classification is still being debated. Mcgovern et al., 2018, for example, used a mock microbial community to evaluate established protocols for rumen 16S rRNA amplicon sequencing, employing primer pairs Pro341F/Pro805R to target the V3-V4 region and 515/806R to target the V4 region. The primers were chosen based on their routine use in previous studies of rumen bacterial and archaeal community analysis. This study emphasized the importance of the number of PCR cycles in microbial community analysis accuracy and specified the number of cycles that appeared to work best with the primers used in their rumen sample study. However, they cautioned that their findings are inconclusive due to the low diversity of the bacterial mock communities used in this study, and they also suggested that such mock microbiome studies be expanded to obtain more tangible representations to support their findings. They also discovered major flaws in previous studies, but confirmed that this supports the need for positive controls in rumen studies and mock microbial communities in such studies. These suggestions will assist researchers in identifying potential errors that may occur during the various steps of the NGS protocol (Mcgovern et al., 2018).

Primers such as ITS-F, ITS4, ITS3, and ITS5 are commonly used for fungal strains (Bellemain et al., 2010), and the entire region, which is approximately 450–700 bp long, has been targeted using traditional Sanger sequencing (Bellemain et al., 2010; Badotti et al., 2017). Bellemain et al. (2010) described the various amplification biases that can occur when ITS primers are used during PCR of different sub-regions with mixed ITS primer templates. This in silico PCR analysis discovered a high proportion of mismatches compared to the targeted sequences, particularly with ITS1-F, resulting in sequence misinterpretation and unavoidable taxonomic biases. Furthermore, they reported similar biases with other commonly used primer combinations, demonstrating amplification biases towards basidiomycetes, such as ITS1 and ITS5, and biases for actinomycetes, such as primers ITS2 and ITS3 and ITS4. Surprisingly, a presumed basidiomycete-specific primer, ITS4-B, only amplified a small fraction of the ITS regions, as significantly observed under standard PCR conditions, casting doubt on identification based on this sequence comparison. As a result, the authors advised that when conducting studies for high-throughput sequencing of environmental samples, one should carefully select the ITS primers. Furthermore, they proposed that different primer combinations or ITS sub-regions be analyzed in parallel. They also suggested looking for alternative ITS primers when dealing with organisms that have previously demonstrated identification biases.

In addition to primer choice, PCR conditions, and the bias that it introduces to data through the hypervariable regions, the cost of sequencing was also a critical consideration for studies seeking to elucidate diverse microbial populations on the traditional sequencing platforms such as Sanger and 454 pyrosequencing. More recently, the emergence and evolution of the NGS platforms have brought about a significant and ongoing reduction in sequencing costs (Mcgovern et al., 2018; Schwarze et al., 2020) with progressive model developments.

**FIGURE 2:**
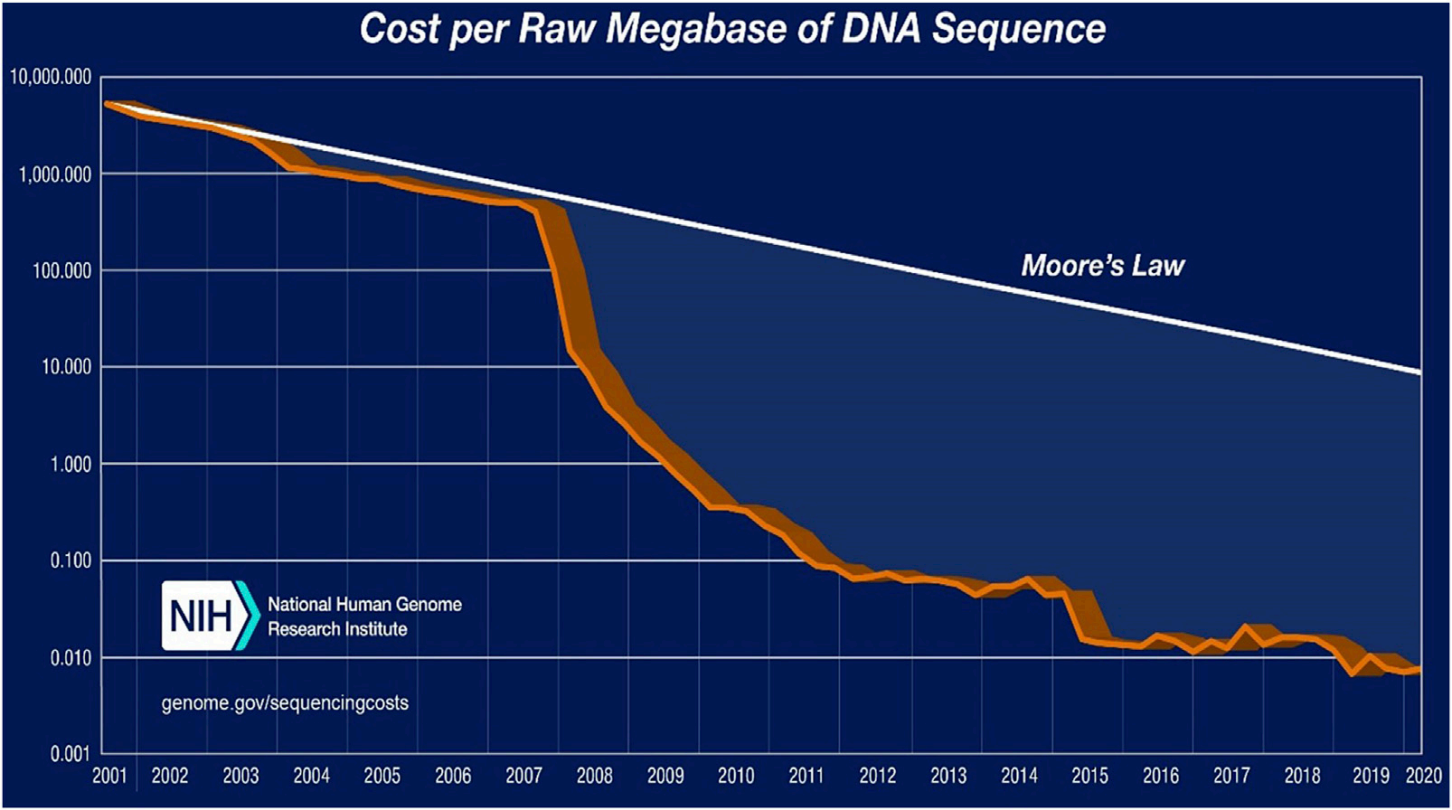
Cost of sequencing per raw MB of DNA sequencing (Wettersrand, 2020)

Remarkably, Sanger sequencing costs around $500 per megabase pair, whereas NGS costs around $0.008 per megabase pair (Wettersrand, 2020). Furthermore, Table 4 and Figure 2 show a significant decrease in sequencing costs since the introduction of NGS, which has eventually mitigated the limitation of sequencing costs to a certain extent and length coverage over time. Several studies on high-throughput sequencing have been conducted over the last few decades, primarily using Sanger sequencing and parallel 454 pyrosequencing (Poinar et al., 2006), followed by other platforms such as the Ion Torrent Personal Genome Machine (PGM), the Illumina MiSeq/HiSeq, and the Applied Biosystems SOLiD systems, which are commonly used in environmental studies (Schuster, 2008; Rodrigue et al., 2010). All of these DNA sequencing technologies produce shorter fragments than Sanger, and SOLiD produces even shorter reads (see Table 4).

**TABLE 4:** The evolution and comparison of sequencing platforms.

Platform	Gen	Amplification method	Read length (bp)	Single pass error rate (%)	Time/run	Cost/million bases ($)	Year	Refs.
Sanger	1^st^	PCR	400–1,000	0.001	0.5–3 h	500	2001	(Schuster, 2008; Liu et al., 2012; Rhoads and Au, 2015)
454 Roche	2^nd^	Emulsion PCR	700	1	23 h	8.57	2006	(Schuster, 2008; Liu et al., 2012; Rhoads and Au, 2015)
SOLiD	2^nd^	Emulsion PCR	2 × 60	5	6 days	0.11	2006	(Liu et al., 2012; Rhoads and Au, 2015)
Illumina HiSeq 2500 (High Output)	2^nd^	Solid phase/Bridge PCR	1 × 36–2 × 125	0.1	29h—6 days	0.03	2008	(Rhoads and Au, 2015; Illumina, 2021)
Illumina HiSeq 2500 (Rapid Run)	2^nd^	Solid phase/BridgePCR	1 × 36–2 × 250	0.1	7–60 h	0.04	2008	(Rhoads and Au, 2015; Illumina, 2021)
PGM	2^nd^	Emulsion PCR	200	1	2–4 h	0.1	2010	Rhoads and Au, (2015)
PacBio SMART, RS II: P6-C4	3^rd^	None (Real-time single-molecule)	1.0–1.5 × $10^{4}\;$ on average	11–15	0.5–4 h	0.4–0.8	2011	(Laver et al., 2015; Rhoads and Au, 2015)
ONT (MiNION)	3^rd^	None (single-molecule nanopore)	2–5× $10^{3}$ on average	38	50 h	6.44–17.90	2015	(Laver et al., 2015; Rhoads and Au, 2015)

While the Illumina platform is economical and permits broad microbial population coverage, it only generates reads that are 70–250 bp long (and a maximum of 300 bp on the Miseq platform), often with reliable, high-quality reads of approximately 250 bp for the forward read and 230 bp for the reverse reads. It is also reported to have no protocol to reliably assemble full genes in microbial community samples (Burke and Darling, 2016). Consequently, this limiting factor results in the reduced ability to capture the hypervariable regions in its entity as these short-reads fail to generate sufficient overlaps between DNA fragments., Furthermore, as a direct result of most bioinformatics techniques’ inability to completely piece together without redundancy the various contigs produced by the platform, low-quality full-length reference sequences are submitted to most databases, affecting even taxonomic representations (Schloss et al., 2016; Pearman et al., 2020). It is worth mentioning that the Nextseq has made tremendous progress in addressing some of the limitations present with the MiSeq sequencing platform. The basic Nextseq 550 produces a maximum output of 120 Gb or single 30X genome with a 400 million maximum number of reads in 12–30 h whereas the Miseq generates 15 Gb with a 25 million maximum number of reads at 4–55 h. However, its major shortcoming is with the maximum read length of 2 × 150 bp and the error rate (<1%) which is not an improvement from the other platforms (Reuter et al., 2015).

Interestingly, the introduction of the long-read sequencing platform such as PacBio by Pacific Biosciences has greatly overcome the limitations that were encountered with the Illumina short reads. In an evaluation study of 16S rRNA amplicon sequencing using the short read Illumina Miseq and near-full length PacBio platforms for phylogenetic analysis of the rumen bacterial community in steers, it was reported that both platforms unearthed similarities in microbial OTUs and species richness, and other metrics. However, the PacBio platform revealed a significant increase in several orders of taxa and showed greater taxonomic classification accuracy. Overall, these findings demonstrate that the data supports a general agreement that longer reads produce finer phylogenetic resolution that may not be obtained by shorter 16S rRNA gene fragments (Myer et al., 2016).

The scarcity of ruminant GIT research using the PacBio sequencing platform is remarkable, considering the aforementioned insights, as provided in Table 4 and the wealth of information impacted by long reads, especially with the acknowledged limited resolutions derived from short read lengths of previous ruminant GIT studies, which have significantly contributed to the under-representation and ultimately the diversity within these communities presents in this ecological niche. A panoramic observation of reports found on NCBI shows that only one study exploits this platform and technology for an *in vitro* investigation of the effects of subacute rumen acidosis on the bacterial community. The study integrated PacBio and Illumina MiSeq amplicons (Brede et al., 2020). Because no studies have used PacBio long read sequencing technology, the short read limitations add to the lack of functional information on many microbial isolates, particularly those in the GIT.

The development of sequencing platforms such as PacBio and the advanced collective technology of ‘omics promises an alternative approach to ameliorating these limitations and, providing greater access to the untapped depth of microbial ecosystems that have adjusted to living under a vast array of adverse conditions. The ‘omics technology not only answers questions such as *“who is there?”* or “taxa” but it also covers the *“what are they doing?”* and *“how are they doing it?”* aspects of microbial communities within a given environment. When utilized in the ruminants’ GIT, the ’omics technology together with the long read sequencing platform could potentially pave the way for enrichment of livestock feed for sustainable utilization and directed manipulation for other industrial purposes and methane abatement strategies (Seshadri et al., 2018).

### Functional genomics as a way forward for unraveling the complexity of ruminants GIT

**FIGURE 3:**
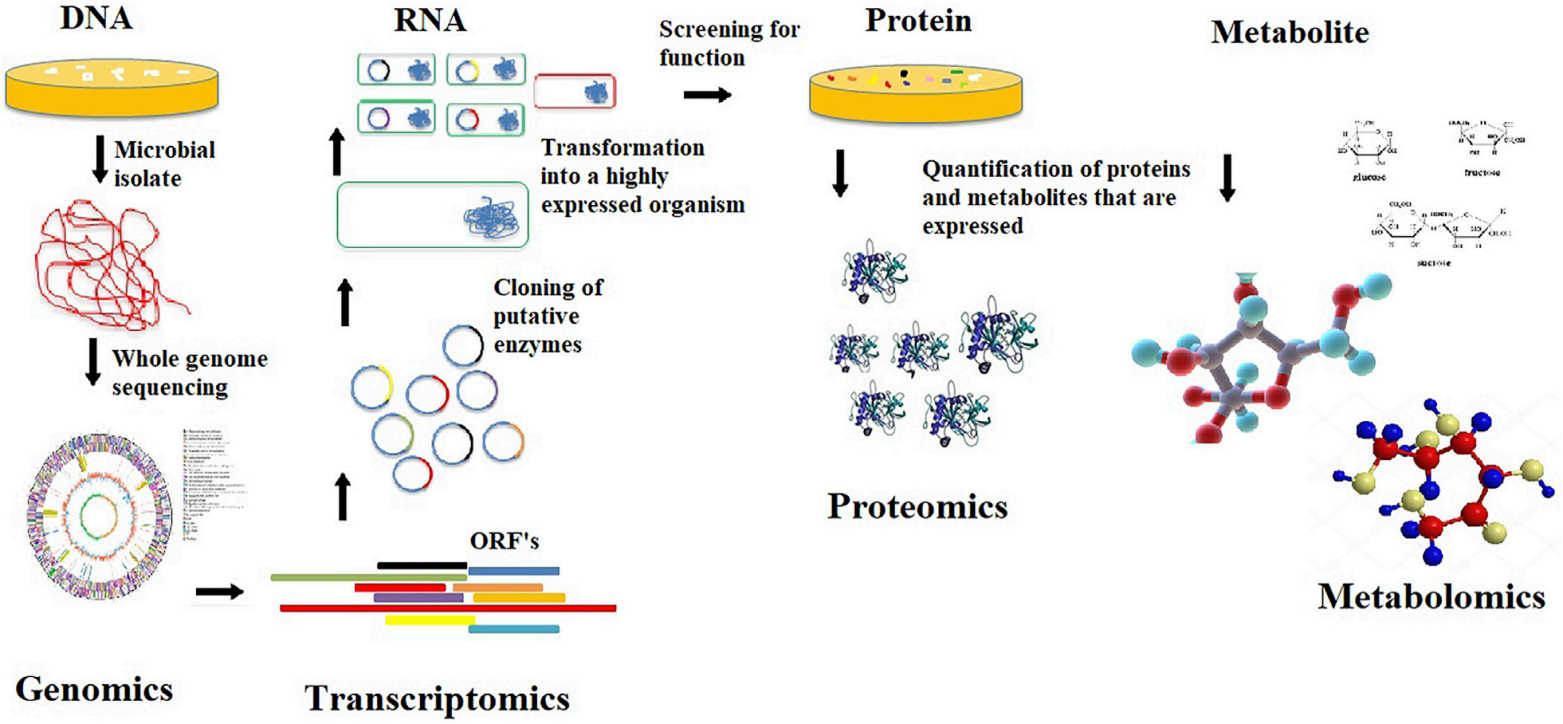
A typical overview of functional genomics.

As an interdisciplinary field of biology, genomics focuses mainly on the structure, function, evolution, mapping, and editing of the genomes of individual organisms. It involves sequencing and analyzing complete genomes from individual microorganisms to assemble and analyze their function and structure. The insight gained from genome sequence information does not directly elucidate the phenotypic traits of an organism. However, studying the functional parts of genome sequences that integrate the other aspects of omics technologies (Figure 3) helps better understand microorganisms’ roles within consortia from unique environments and paves the way forward to improving their bio-products for industrial and environmental applications (Hardison, 2003). Functional genomics differs from metagenomics, which utilizes sequencing of the entire environmental sample comprising multiple genes from various microorganisms and has been reported to potentially capture about 99% of microbial genes in a given sample (Handelsman, 2004).

Furthermore, metagenomics studies integrate library construction to bridge the gap between microbial communities’ structures and their functional roles. Cheng et al. (2012a) constructed a metagenomic library of Chinese Holstein cow rumen microorganisms using the *E. coli* EPI300 and pcc2FOS vectors and screened them for novel gene clusters on ethyl ferulate (FAE-SH1). Functional expression of this novel gene that displayed 56% similarity to the previously identified methyl esterase enzyme was achieved in *E. coli* BL21 (DE3). Moreover, FAE-SH1 exhibited a broad resistance to proteases and was reported to enhance the release of ferulic acid from wheat straw with cellulase, β-1, 4-endoxylanase, β-1, 3-glucanase, and pectinase. Subsequent studies using the same library used another novel gene (Xyln-SH1) that demonstrated a 44% similarity to the previously identified glycoside hydrolase from *Clostridium thermocellum* ATCC 27405 (Cheng et al., 2012b). These two studies highlight the unlimited wealth of biocatalyst availability in the GIT environment. Furthermore, their usefulness in ligninolytic biomass degradation implies the worthwhile continuation of bioprospecting in various species of ruminants for potential microbial candidates in the production of enzymes necessary for improved health effects of food and forage (Cheng et al., 2012a) and application in biomass pre-treatment for industrial applications. A metagenomic library of a Cashmere goat rumen microbiome was constructed from DNA fragments ranging from 50 to 150 kb using the BAC vector (pCC1BAC) and *E. coli* EPI300. The authors reported having acquired eight clones with amylase activity (Zhang and Chen, 2014). Bi-function recombinant proteins have been reported from bovine (Rashamuse et al., 2013) and goat rumen (Cheng et al., 2016). Novel enzymes, bioactive and biosynthetic pathways (Coughlan et al., 2015) with potential applications in the food and pharmaceutical industries have been discovered through functional metagenomics. For example, three novel carboxylic hydrolases and alkaline serine proteases (Biver et al., 2013; Biver and Vandenbol, 2013) have been identified from forest soil through microbial activity screening procedures of metagenomic libraries. Moreover, Coughlan et al. (2015) reported novel antimicrobial, anti-infective, pollutant degradation, and antimicrobial resistance genes discovered through functional metagenomics.

While this approach continues to provide insight for advanced biotechnological applications in industrial and environmental applications and reveals significant progress in the understanding of the ruminants’ GIT microorganisms’ role on a genetic level, particularly for novel gene mining, it suffers from a credible limitation in the generation of a large number of unassigned sequences within microorganism communities. Thus, this review expresses concern in light of the gaps observed in the current approach to the use of metagenomics as they relate to unidentified sequences and the inability to link them to known microorganisms and vice versa, which is a recurring limitation in metagenomics studies. Thus, functional genomics is a potential approach in studies involving genetic materials recovered from natural samples, bridging the gap between sequence availability discrepancies in the absence of reference cultures.

Downstream approaches such as transcriptomics, proteomics, and metabolomics are used in functional genomics to determine the conditions under which these microbial genomes play a critical role at various levels (Denman and Mcsweeney, 2015). Since 1981, complete sequence genomes of bacteria have been reported (Anderson et al., 1981). Because of the rapid intensification of the scope, the turnaround time of genome sequencing projects, and the constant cost reduction, genomes have continued to be sequenced at an exponentially increasing rate. In 2003, one of the first significant studies completed the genome and sequence analysis of *Wolinella succinogenes*, the first bacteria sequenced from the GIT of ruminants (i.e., bovine) (Baar et al., 2003). Although the purpose of this study was not to investigate function in the GIT of ruminants, but rather to understand the origin and emergence of pathogenic bacteria in humans, it did take advantage of this black box opportunity. It prompted the realization that microbial genome sequences could aid in understanding of microbial populations’ phenotypic and genotypic potential within ruminant GIT.

Strides have been made to promote advancement in ruminant genome research, including the establishment of the North American Consortium for Genomics of Fibrolytic Ruminal Bacteria to provide the annotated sequences of fiber-degrading bacteria within the GIT of ruminants, and this has ultimately led to the sequencing of *Fibrobacter succinogenes*, *Ruminococcus albus*, and *Prevotella ruminicola* genomes (Jun et al., 2007; Brumm et al., 2011; Leahy et al., 2013). Subsequent sequencing of ruminant microbial genomes studies underpinning the knowledge of their role in polysaccharide degradation, short-chain fatty acid production, methanogenesis pathways, and assigning specific taxa to functions have been presented (Seshadri et al., 2018). This was massively achieved with the collaborative work of the Hungate1000 project (www.Hungate1000.org.nz), involving 60 research scientists within 16 organizations in nine countries, to produce a reference set of 1,000 microbial genome sequences by sequencing the genomes of culturable bacteria and methanogenic archaea, which also includes representative cultures of anaerobic fungi and ciliate protozoa from the GIT of ruminants. The American Type Culture Collection (ATCC), Culture Collection University of Göteborg (CCUG), Leibniz Institute DSMZ-German Collection of Microorganisms and Cell Culture (DSMZ), Japan Collection of Microorganisms (JCM), Belgian Co-ordinated Collections of Microorganisms (BCCM/LMG), and the National Collection of Type Cultures (NCTC) are among the major culture collections that have contributed to the Hungate1000 project. There are about 89.02% of entries of genomes whose culture collections are not specified, followed by 9.76% from the DSMZ, 0.98% from ATCC, and 0.24% from LMG (Seshadri et al., 2018). Before this project was initiated, only 15 reference genomes were available to the scientific community. However there are currently 431 reference genomes available of bacteria and archaea from the GIT of various ruminant origins (https://genome.jgi.doe.gov/portal) (Seshadri et al., 2018).

The Hungate1000 project is primarily dominated by genomes from the phyla *Firmicutes*, *Bacteroidetes*, *Actinobacteria*, *Proteobacteria*, *Euyarchaeota*, *Spirochaetes*, *Synergisters*, *Fibrobacteres*, and *Fusobacteria*. Some of which are involved in the substrate utilization of functional groups (xylan, starch, cellulose, lipids, and protein, among others), breakdown of end-products (butyrate, propionate, lactate, succinate, e. t.c), and metabolic pathways such as the Enolase and Entner–Doudoroff pathway (Seshadri et al., 2018). The majority of the genomes presented in the Hungate1000 project are from the cow’s (262) and sheep’s (61) rumen, followed by moose with eight entries and three genomes from the deer and goat’s rumen. Although the Hungate genome catalog allows robust comparative genome analysis, which brings forth the understanding of the breakdown of plant polymers such as lignocellulose to soluble end products, the project is still ongoing as several important taxa have not been captured, particularly members of the order Bacteroidales (Seshadri et al., 2018). However, the resulting data has shown a maximum potential to unravel the complexity of the ruminant’s GIT function, feed conversion efficiency, methanogenesis, and plant cell wall degradation (Seshadri et al., 2018). Moreover, the Hungate1000 can bridge the gap between the ruminant GIT metagenomic and metatranscriptomics sequence datasets and bring insight into the GIT microbial phylogenetic diversity through genome sequencing of microorganisms that have not yet been captured.

One could argue that functional genomics is limited because environmental samples must be grown in a lab to obtain pure cultures. However, there appears to be a revival of old microbiology techniques that have been modified to accommodate fastidious microorganisms (Lagier et al., 2015). Prof. Raoult’s research group pioneered the re-imagination and integration of the high-throughput culture method, known as culturomics, in 2012 to facilitate the study and taxonomic identification of complex human gut microbiota (Lagier et al., 2012). It takes into account multiple culture conditions in order to promote the growth of fastidious microorganisms from the human gut. Although the technique was originally designed to identify novel bacterial species in the human gut microbiota, it has recently been applied to other microbial environments such as the human vagina (Diop et al., 2018; Diop et al., 2019), urinary microbiome (Dubourg et al., 2020), and mammalian gut (Pereira and Cunha, 2020). The culturomics approach includes sample segmentation and diversification based on various cultural conditions. It can accomplish this by incorporating the use of cultural conditions that suppress the growth of the majority of the population, which were most likely identified in the past due to their amenable disposition to most environmental conditions. As a result, the culturomics approach promotes the growth of fastidious microorganisms, which are frequently present in lower concentrations (Lagier et al., 2015; Lagier et al., 2016). Recently, some authors, including Masucci et al. (2017) and Bilen et al. (2018), have emphasized the importance of the modern culturomics approach in elucidating the so-called ‘unculturable’ microbial communities. They emphasize that the difficulty in cultivating these “uncultivable organisms” is easily overcome if the proper conditions and tools are used (Lagier et al., 2015; Bilen et al., 2018).

In an attempt to explore the potential of culturomics to characterize the rumen microbiome, Zehavi et al. (2018) provided significant insights into the culturomics of Israeli Holstein Cows. Their study found that the cultivation experiment captured only 23% of all OTUs in the rumen microbiome. However, they were able to identify factors that affect microbial richness on solid media by exploring various media compositions and showing a relationship between this factor and the likely increase of the number of cultured OTUs by as much as 40%. Also, it was noted that sample dilution had the most potent effect on increasing the microbial population richness on the plates. At the same time, abundance and phylogeny were the main factors determining the cultivability of rumen microbes. However, they cautioned that the likelihood of multifactorial traits is partly responsible for limited cultivability. Nevertheless, it was remarkable that a significant portion of the cultured OTUs obtained belonged to a rare biosphere and could not be detected from the established microbiome, even after it was compared to 38 rumen microbiome samples. In summary, their study’s unique dimension and complexity demonstrate the need for further investigation of culturomics and metagenomics and reveal the need for extensive research in this environment.

As a result, by capturing credible sample sizes of these organisms, providing data from combined physiological studies, and verifying predictions derived from genome sequence data, the integration of all components of ‘omics technologies within the framework of research designs and methodologies will gradually alleviate previous limitations (Lagier et al., 2012; Lagier et al., 2015; Lagier et al., 2018). Furthermore, the forward-backward strategy, which employs a culture-independent approach, may improve the design of culture media based on precepts derived from metagenomics data, which better promotes the proliferation of these fastidious organisms present in ruminants’ GIT, thereby mitigating the cultivation challenge.

### Bioinformatics tools and their usefulness in the generation and interpretation of data

The availability of bioinformatics tools has increased the appeal and accessibility of DNA sequencing. However, the sheer number of available tools can make determining which one is best for a given project difficult. These software programs extract large amounts of complex biological data from a pool of molecular biology databases and generate readable sequences and structured datasets. Tax4fun, FaproTax, CowPI, and PICRUSt are some bioinformatics tools that reveal predictive analysis of functional microbiome profiles (Sun et al., 2019). CowPi and PICRUSt are the most widely used and compared bioinformatic tools for robustness and efficiency, particularly in predicting rumen microbiome samples. They do, however, have benefits and drawbacks, just like any other tool. PICRUSt can predict function from 16S marker gene sequences and shotgun metagenomic sequences, as previously stated. However, it does not work well on shotgun metagenomic sequences if there is host contamination and non-microbial DNA dominance in samples or if there is inadequate community biomass (Douglas et al., 2020a).

When PICRUSt was first established, it produced predictions limited by the genomes that were accessible at the time, which were heavily biased towards microbes connected with human health and biotechnological utilization. Furthermore, the input sequences for PICRUSt and its standard workflows must be OTUs table with IDs present in the GreenGenes database created by closed-references OTU (Wilkinson et al., 2018; Douglas et al., 2020b). As a result, the predefined PICRUSt workflow is now incompatible with sequence denoising methods that generate amplicon sequence variants (ASVs) rather than OTUs due to the restriction to reference OTUs. With recent development on PICRUSt, available as PICRUSt2, it is expected that optimizing genome prediction would improve functional prediction accuracy. As a result, the PICRUSt2 algorithm includes steps that optimize genome prediction, such as placing sequences into a reference phylogeny rather than relying on predictions limited to reference OTUs, predicting pathway abundance more stringently based on a larger database of reference genomes and gene families (Douglas et al., 2020a).

Sun et al. (2019) evaluated PICRUSt’s accuracy on seven datasets containing human (2), non-human animal (gorilla, mouse, and chicken), and environmental (2 soil samples) sequences. It was discovered that the Spearman correlation is not a reliable measure of gene content prediction accuracy. PICRUSt results for inference showed greater consistency with metagenome sequences in human samples than in non-human samples, in contrast to Spearman correlation. Furthermore, when the difference in performance between PICRUSt and metagenome sequencing for inference in different functional categories was investigated, PICRUSt performed differently for each functional category (Sun et al., 2019). Although, it was not specified which version of PICRUSt was used in this study, the findings suggest that developing tools tailored to specific environments will be useful for better predicting functional microbial profiles to induce accuracy (Wilkinson et al., 2018; Sun et al., 2019).

One of the exciting examples of advancements to generate environmental-specific datasets is the development of CowPI, a focused version of the PICRUSt tool for functional inference tool specific to the rumen microbiome. The Global Rumen Census and Hungate 1,000 project provides CowPI’s datasets communities to aid in discovering functional rumen microbiomes. As a result, the KEGGREST (Tenenbaum, 2017) package in R was used to extract Kyoto Encyclopedia of Genes and Genomes (KEGG) orthologs (KO’s) IDs and construct a frequency count of KO IDs found in each Hungate1000 genome using the unique Uniprot IDs as a starting point (Wilkinson et al., 2018). CowPI produced better estimates than PICRUSt when predicting functional profiles in the bovine environment as a result of these parameters. Although both PICRUSt (especially PICRUSt2) and CowPI provide insights that would otherwise avoid the inclusion of rather expensive metatranscriptomic exercises, Wilkinson et al. (2018) caution against relying solely on predictive tools in our comprehensive investigation and understanding of genome sequences.

Nevertheless, PICRUSt has been used to predict the function profiles of genes involved in metabolic processes such as carbohydrates, lipids, amino acids, genetic and environmental information processing, and human diseases (Sun et al., 2019). In another study, PICRUSt was applied to 16S rRNA marker genes to predict the co-digestion strategies of cow, horse, and pig manure for biogas production. Across all samples, they discovered 135 KOs responsible for amino acid, carbohydrate, energy, lipid, and xenobiotic metabolism (Ijoma et al., 2021). PICRUSt has also been used in the functional profiling of crude-oil-polluted soil (Chikere et al., 2019) and epiphytic bacterial communities from rocky intertidal seaweeds (Chikere et al., 2019; Selvarajan et al., 2019). Meanwhile, CowPI was used in a lamb comparison study to uncover functional profiles of the rumen microbiome, which revealed the various predictions across all three tests (Trabi et al., 2019). The CowPI was also used to profile goat rumen microbial colonization, confirming that goat rumen maturation occurs in three stages (Zhang et al., 2019). This research paves the way for further advancements in developing feed for the ruminant production sector. Researchers recently used PICRUSt and 16 sRNA to predict co-digestion strategies of animal manures for biogas production (Ijoma et al., 2021). Both PICRUSt and CowPI research, particularly CowPI, which is unique to the rumen environment, promises to open up a whole new arena in bridging the gap between phenotypic and genotypic data. As a result, more functional studies of rumen microbiomes will be added to the CowPI database. Because these tools are so new, it is understandable why there is so little functional data and application of rumen microbiomes.

### Major limitations of ’omics technology

The use of recombinant DNA technology, such as cloning, is an essential component of functional genomics, including omics technology, particularly when used in the search for novel enzymes for overexpression. Because enzymes are frequently unsuitable for use as industrial biocatalysts in their natural state, cloning has revolutionized the expression of enzymes for industrial use (Porter et al., 2016). However, even when microbial enzymes are used as whole microbial cells for biomass bioprocessing, overproduction or high titer values are nearly impossible to obtain in the native microorganism due to feedback inhibition because the organism can only produce the amount of enzyme required for survival, necessitating additional modifications (Porter et al., 2016). Among the additional modifications is the transformation of an engineered overexpression organism with putative enzyme ORFs. However, as discussed in Section 4.1.1, while cloning has increased the ease of production, robustness, and titre value of by-products, gene expression in heterologous systems may imply differences in chaperones, posttranslational modifications, and codon usage, all of which have the potential to interfere with expression patterns (Pourmir and Johannes, 2012). Such issues continue to present challenges in the application of ‘omics technologies. Thus, it is critical to consider the selection of a suitable host organism for cloning in order to functionally express putative enzymes (Pourmir and Johannes, 2012). Furthermore, exposing DNA or plasmid to UV light has the potential to damage the DNA, resulting in false or empty recombinants. However, the latter challenge can be overcome by staining agarose gels with crystal violet instead of ethidium bromide to visualize DNA and improve cloning efficiency (Rand, 1996; Turgut-Balik et al., 2005).

## Concluding remarks

The main objective in applied biocatalysis is to yield large quantities microbial enzymes with desired attributes using inexpensive but efficient approaches. Hence, the progressive demand for these enzymes of industrial value has stimulated the bioprospecting of unusual or unique sources. This has driven advanced molecular techniques to meet the challenge of improving enzyme technology, which adds value to commerce, industry, and the environment. One such unique environment is ruminants’ gastrointestinal tract, which is home to fastidious microorganisms such as bacteria, fungi, protozoa, and viruses, some of which secrete various enzymes, including lignocellulosic enzymes, among others, as a result of a mutually beneficial relationship with the animal. Due to the nature of this environment (anaerobic, balanced pH, temperature, and feed type), accessing these microorganisms has remained a challenge for decades. This review highlighted the challenges and limitations that have led to the insufficiency in functional genomics studies, data, and applications specific to bio-prospecting research in the microbiome of ruminants. However, the review delved into other extreme environments with similar challenges to reach these objectives. It can be summarised that factors such as the utilization of culture-dependent approaches, targeted hypervariable regions (16S, 18S, and ITS) studies, cost of sequencing, and read lengths of sequences have contributed to the dearth in functional sequence data that will move application-based work forward. The empirically-based investigation provided by this review provides that the pragmatic shift to integrate ’omics technologies routinely into most research and development around ruminant GIT bioprospecting will greatly benefit the progress toward industrial biocatalysis.
