# Supplementary material for: Insufficiency in functional genomics studies, data, and applications: A case study of bio-prospecting research in ruminant microbiome
Source: Front Genet. 2022 Aug 31;13:946449. doi: 10.3389/fgene.2022.946449 (PMC9472250; doi:10.3389/fgene.2022.946449)
Supplement: Supplementary file 1 [file DataSheet1.docx]

**Insufficiency in Functional Genomics Studies, Data, and Applications: A Case Study of Bio-Prospecting Research in Ruminant Microbiome**

Kgodiso J. Rabapane^1*^, Grace N. Ijoma^1^, & Tonderayi S. Matambo^1^

*^1^ Institute for the Development of Energy for African Sustainability (IDEAS)*

*University of South Africa’s College of Science, Engineering, and Technology*

Cnr Pioneer and Christian De Wet Roads, Private Bag X6, Florida, 1710, South Africa

** Corresponding author*: Email: kgodiso93@gmail.com. Telephone: +27 11-6709049

## Supplementary Information

**Table S1: Targeted sequence studies**

| **Ruminant** | **Approach** | **Sequencing Platform** | **Read length** | **Primer set** | **Diet** | **Strain/Dominant Phyla** | **Enzyme** | **Growth conditions** | **Origin** | **Year** |
| --- | --- | --- | --- | --- | --- | --- | --- | --- | --- | --- |
| Cattle | Total DNA |  | 1060 bp | 27F/1492R | Haylage, corn and silage | G+C Gram-positive bacteria | - | - | Canada | 1998 |
| Reindeer | Culture-based | ABI Prism® 3130XL Genetic Analyzer |  | 27F/1522F | Mixed diet dominated by  lichens | *Eubacterium rangiferina* (usnic acid-resistant) | - | 39°C/24hrs | Norway | 2008 |
| Svalbard Reindeer | Total DNA | ABI 3130x1 Genetic  analyzer | ~1200 bp | Met86F/Met1340R(16S)- 968F/1401R (16S)-316F/539R(18S) | - | Diversity | - | - | Norway | 2009 |
| Yak | Culture-based | ABI PRISM 377XL DNA  sequencer | 1547 bp | 27F/ 1541R | - | *Cellulosilyticum ruminicola* | *-* | An/ 38^o^c/48hrs | China | 2010 |
| Goat | Total DNA |  | 400 bp | 27F/1492R- 109F/915R | - | Bacteroidetes and Firmicutes | - | - | Brazil | 2011 |
| Holstein Cows | Total DNA | 454 FLX Titanium | 460 bp | 343F/ 784R | Starch and fat | Microbial diversity | - | - | France | 2012 |
| Surti Buffalo | Total DNA | ABI Prism 310 Genetic analyser |  | * | green fodder bajra, mature pasture grass and concentrate  (20% CP, 65% TDN) | unidentified protozoa, *Kinetofragminophorea* | - | - | India | 2013 |
| Deer | Culture-based |  | 519-597 nt | rpsA-rpIB | - | Actinomyces sp. c10 | Xylanase | An/39^o^c/ 72 hrs | CzechRepublic | 2017 |

* protozoa-specific forward primer (5’-ACTTTCGATGGTAGTGTATTGGACTAC-30)/Eukarya-specific reverse primer (5’-ATGATCCTTCTGCAGGTTCACCTAC-3’

**Table S2: Sequence-Based Metagenomics**

| **Ruminant** | **Sequencing Platform** | **Read length** | **Diet** | **Dominant Phyla** | **Predicted protein** | **Origin** | **Year** |
| --- | --- | --- | --- | --- | --- | --- | --- |
| Mehsani buffalo | Ion Torrent PGM | 146-180 bp | Dry roughage, concentrate, green roughage | *Firmicutes* | Carbohydrate and protein metabolism | India | 2014 |
| Buffalo | Ion Torrent PGM | - | Dry and green roughage | *Bacteriodetes* | CAZymes | India | 2014 |

**Table S3: Functional-Based Metagenomics**

| **Ruminant** | **Vector** | **Library host** | **Library size** | **Sequencing Platform** | **Read length (bp)** | **Diet** | **Similarity (%)** | **Enzyme** | **opt. conditions** | **Origin** | **Year** |
| --- | --- | --- | --- | --- | --- | --- | --- | --- | --- | --- | --- |
| Bovine | pCC2FOS | *E.coli* EPI302 | 70 000cfu | 454-pyrosequencing | 1677/1666 | N/A | 75% endo-1,4-b-D-glucanase [unidentified microorganism (ABX76045.1) | Cellulase, Xylanase (cel5A and cel5B) | pH 9.0 and 65°C | South Africa | 2012 |
|  |  |  |  |  |  |  |  |  |  |  |  |
| Holstein cow | pCC2FOS | *E.coli* EPI300 | N/A | N/A | 789 | N/A | 44% glycoside hydrolase [*Clostridium thermocellum* (ATCC 27405) | novel feruloyl esterase (FAE-SHI) | pH 8.0, 40°C | China | 2012 |
| Holstein cow | pCC2FOS | *E.coli* EPI300 | N/A | N/A | 1056 | N/A | 44% glycoside hydrolase [*Clostridium thermocellum* (ATCC 27405) | novel xylanase (Xyln-SH1) | pH 6.5 and 40°C | China | 2012 |
| Goat | pCC1BAC | *E.coli* EPI301 | 14 400 clones | N/A | N/A | N/A | N/A | amylase | N/A | China | 2014 |
| Korean black goats | pCC1FOS | *-* | 115200 clones | ABI 3730 automatic sequencer | 2577 | - | 77% endo-β-1,4-glucanase (*Fibrobacter succinogenes)* | KG37 Cellulase | pH 5.0 and 20–50 °C | South Korea | 2016 |
| Korean Black Goats | pCC1FOS | *E. coli DH5α* | 115200 clones | Sanger sequencing and pyrosequencing | 963 | rice straw and mineral supplements | 55% Hypothetical protein  (*Eubacterium cellulosolvens*) | KG35  endo-β-1,4-glucanase | pH 6-7 and 30-50°C | South Korea | 2017 |
|  |  |  |  |  |  |  |  |  |  |  |  |
